# Laboratory risk management for INR alert values

**DOI:** 10.1038/s41598-025-16944-z

**Published:** 2025-08-29

**Authors:** Oana Roxana Oprea, Ana-Maria Fotache, Corina-Maria Rus, Minodora Dobreanu

**Affiliations:** 1 Department of Laboratory Medicine, Faculty of Medicine, George Emil Palade University of Medicine, Pharmacy, Science, and Technology of Targu Mures, 540142 Targu Mures, Romania; 2 Clinical Laboratory, Emergency County Clinical Hospital of Targu Mures, 540136 Targu Mures, Romania

**Keywords:** INR (The international normalized ratio), Alert value, Sysmex CS-2500 instrument, Tertiary care hospital, Biochemistry, Health care, Medical research

## Abstract

The International Normalized Ratio (INR) is a laboratory test used to monitor and adjust medication with vitamin K antagonists. The alert values of the INR obtained in the laboratory must be managed efficiently to provide the best management for the patient. The aim of the study was to evaluate the risk levels associated with INR alert values for the laboratory in a tertiary care hospital and compare the findings with the established benchmarks. The study was conducted from September 2023 to August 2024 at the Clinical Laboratory from Emergency County Clinical Hospital of Targu Mures. Both risk levels associated with the occurrence of alert values for INR > 4.5 and underlying causes of these values were assessed. Plasma samples collected in all wards were analyzed using Sysmex CS-2500 instrument. The sample’s journey through the laboratory was analyzed. The risk was assessed and a daily probability of alert value occurrence for INR was calculated. A total number of 42,799 plasma samples were analyzed, out of which 347 (0.81%) had INR alert values and were collected from 246 patients, some having more than one sample collected. This study showed that INR alert values > 4.5 represent an Unacceptable risk level, with an expected daily occurrence of λ = 0.95 (61% probability of ≥ 1 event per day). The consequence is critical in the risk assessment matrix that corresponds to a frequent probability. The laboratory considered the risk of INR values > 4.5 to be unacceptable both as frequency and consequences. Incomplete documentations of phone communications and resampling delays (> 6 h in 30% of the samples) were identified as potential failure modes. Therefore, laboratories are expected to implement strict control measures to effectively mitigate this risk.

## Introduction

The International Normalized Ratio (INR) is a standardized laboratory test used to monitor anticoagulant therapy with vitamin K antagonists (eg. Warfarin) and to guide dosage adjustment^1^. The INR values also recognized as independent predictors of mortality in cancer patients^2^.

Alert (also called critical values) indicate potentially life-threatening conditions and require prompt corrective action to ensure patient safety^3^. The selection of alert values varies between laboratories, depending on guidelines, agreements with the clinicians and patient population patients. Although studies focusing on the occurrence of INR alert values are limited, a tertiary hospital study reported a frequency of 0.61%^4^.

Risk management in the clinical laboratory is an essential component of quality management system and is mandated by ISO 15189:2022 Standard^5^ which emphasizes a continuous cycle of risk identification, quantification, mitigation and monitoring^5^. The acceptability of a given risk depends on both its probability of occurrence and the severity of its consequences^6^. These consequences may refer to the patient, clinician or the laboratory itself as an organization.

The aim of the study was to assess the risk associated with INR alert values for in a tertiary care hospital laboratory by applying a quantitative probability model within the ISO 15189:2022 framework. Operational metrics including the timing of second sample collection and documentation of phone communications, were also evaluated to provide benchmarks for future risk mitigations.

## Material and methods

A retrospective study was conducted from September 2023 to August 2024, at the Clinical Laboratory from Emergency County Clinical Hospital of Târgu Mureș (a tertiary care hospital) with ED (Emergency Department) and ICU (Intensive Care Unit) wards. The study was approved by the Ethics Committee (no. 2045/23.02.2024) of the Emergency County Clinical Hospital of Targu Mures and informed consent was waived because of the retrospective design of the study.

### Population

Plasma samples collected in all wards were analyzed using Sysmex CS-2500 instrument. Data were extracted from the laboratory information system (LIS). Demographic data (age, sex), the requesting ward (ED, ICU, or other) and the patient’s primary diagnosis, were recorded from the Laboratory Information System (LIS).

### Risk assessment

To quantify the daily probability of INR alert values, we applied a Poisson probability model. The expected daily number of alert values (λ) was calculated as: λ = ndaily*p, where ndaily = Total samples/Days and represents the mean daily number of samples and p = Alert samples/ Total Samples and is the probability of an alert sample. The probability of having at least one INR alert value per day was calculated as: *P* (≥ 1) = 1 − ℯ^(−λ)^.

Severity and probability categories were defined in accordance with ISO 15189:2022 risk management principles and adapted from laboratory risk assessment literature^5–8^. These are presented in Table 1.

**Table 1:** Severity of consequences and probability of occurrence.

Severity level	Definition	Probability level	Definition
Negligible	No clinical impact; does not affect patient care	Frequent	> 50% chance of occurrence per day
Minor	Temporary inconvenience; no treatment or follow‑up required	Most likely	10–50% chance of occurrence per day
Major	Requires clinical monitoring or minor intervention	Likely	1–10% chance of occurrence per day
Critical	Requires urgent medical intervention (e.g., vitamin K, plasma transfusion)	Rare	< 1% chance of occurrence per week
Catastrophic	High risk of death or permanent disability	Unlikely	< 1% chance of occurrence per month

Probability thresholds were adapted from ISO 31000:2018^8^, CLSI EP23-A^7^, and published laboratory risk assessment frameworks^5,6^. Laboratories are encouraged to adapt these thresholds to their own patient populations and organizational contexts.

### Operational matrix

For patients with repeated INR testing, the time interval between the receipt of the first sample and the collection of a second sample was recorded. Cases in which alert values were communicated by phone were also recorded, when explicitly recorded in laboratory records.

Risk assessment was performed using 5 × 5 matrix, assigning risk level based on the calculated probability and the defined severity of clinical consequences. The data were analyzed in Microsoft Office Excel 2016.

## Results

A total of 42,799 plasma samples were analyzed out of which 347 (0.81%) had INR alert values and originating from 246 unique patients. Out of the these, 231 results were between 4.5–7 and 116 results were > 7.

The average number of samples analyzed per day was 117.3. The expected daily number of INR alert values was λ = 0.95, corresponding to a 61% probability of observing at least one such value per day. In the risk matrix, this corresponds to a Frequent probability with a Critical consequence, resulting in an Unacceptable risk level. The risk matrix is shown in Table 2.

**Table 2:** The risk assessment matrix according to the frequent probability.

Severity/Probability of occurrence	Neglectable	Minor	Major	Critical	Catastrophic
Frequent	Unacceptable	Unacceptable	Unacceptable	Unacceptable*	Unacceptable
Most likely	Acceptable	Unacceptable	Unacceptable	Unacceptable	Unacceptable
Likely	Acceptable	Acceptable	Acceptable	Unacceptable	Unacceptable
Rare	Acceptable	Acceptable	Acceptable	Unacceptable	Unacceptable
Unlikely	Acceptable	Acceptable	Acceptable	Acceptable	Acceptable

Significant values are in bold.

Most alert values, (292/347;84%) were obtained from patients older than 60 years; only 7 (2%) in patients under 18. The majority (199/347;57%) were from the Emergency Department with 25 (7.2%) from ICU patients.

For the 42 patients that had a second sample collected, the mean time to repeat sampling was 5.3 h (median 4.2 h; range 0.8–16.5 h). Most samples (23/42) were collected in 2–6 h from the initial collection; 6/42 in less than two hours but 13/42 samples were collected after more than six hours. Although all alert values were flagged in the LIS ensuring no critical results were missed, only 7 cases were explicitly documented as communicated by phone. The true number was likely higher due to incomplete manual recording.

In 30/ 246 (12%) the main cause for ED presentation was bleeding (type of bleeding is shown in Table 2) and six patients presented themselves for INR above 7 at screening tests performed in outpatient units but without bleeding.

The main underlying causes for these values are shown in Table 3.

**Table 3:** Types of bleeding.

Bleeding type	No. of patients^30^
Nose	7/30
GI-lower [melena]	12/30
Gum bleeding	2/30
GI-upper	2 [1 patient was known with F V Leiden mutation]/30
Conjunctival bleeding	1/30
Hematoma	5/30
Hematuria	1/30

One of the two patients with upper GI bleeding was known with FV Leiden mutation.

Out of these, 23 patients received treatment with fresh frozen plasma, 19 received vitamin K and 9 received both.

## Discussions

This study assessed the risk associated with INR alert values in a tertiary hospital laboratory using the ISO15189:2022 risk management framework. The analysis demonstrated that INR > 4.5 represent an Unacceptable risk level due to their frequent occurrence and their association with bleedings requiring medical intervention. The consequence for the patient was classified as Critical in accordance with ISO15189:2022 definitions. Although such events occur routinely in the laboratory, their frequency does not minimize their clinical severity. Compared with previously published studies of 0.61% in a tertiary hospital^4^, our study found a slightly higher proportion (0.81%) of INR alert values. The difference may be partially explained by lower threshold of 4.5 used in our study. This threshold was established in accordance with the clinicians from our ED. In the literature the value of 5.0 is considered as an evidence-based alert value but laboratories should consider this value as a starter list and tailor the list of alert values according to their needs^3^.

The consequences for the patient may not be similar in all hospitals or laboratories. In the population of our study, all patients needed medical attention since the majority of INR alert values were obtained in patients from ED (57%) and 30 patients (12%) had bleedings as the main presentation cause. In the University Hospital Aga Khan, 25 out of the 240 patients (10.6%) included in the study had bleeding at presentation^4^.

One of the consequences, beyond medical care needed that in some cases, is sample recollection. The re-collection of samples may have different reasons: for some patients the reason behind this is confirmation of the initial value and for others a re-check after a medical intervention. In our sample population, 17% of the patients had a sample re-collected and most of them (23/42) in less than six hours from the initial sample.

The rationale for this study was to evaluate not only the probability occurrence but also to enable the laboratory to better allocate resources to manage these values. The occurrence of alert INR values triggers a series of events in the laboratory that need clear guidelines and SOP’s similar for all alert values^3^. In some studies^1,3,9^ the time spent by the laboratory staff to communicate such values was evaluated and the use of automated alerts systems and direct communication was compared^10,11^. In our hospital, all alert values were documented in the LIS but only seven were documented as direct communication. The different factors (like the occurrence of alert values during working hours and outside working hours) that may influence communication time was also assessed^12,13,14^. Since this study was conducted in an Emergency Laboratory, such differences are not expected but the low number of documented communications is likely due to staff diligence on documenting their actions.

Although differences in practice impacts the way clinicians respond to alerts, the use of AI to support these actions was also assessed ^14,15^, and it has been found that at least for alert values in duplicate measures the computer algorithm support could have averted more than 1900 alerts.

By integrating a quantitative probability model with ISO-based risk classification and operational benchmark, the study provides a methodology that other laboratories may use and adapt for the risk management cycle of risk identification, quantification, mitigation and monitoring.

## Conclusions

This study showed that INR alert values > 4.5 represent an Unacceptable risk level, with an expected daily occurrence of λ = 0.95 (61% probability of ≥ 1 event per day). Although frequent, these values retain clinical severity as they often require medical intervention. Incomplete documentations of phone communications and resampling delays (> 6 h in 30% of the samples) were identified as potential failure modes. To mitigate the risk laboratories should adopt automated alert systems and prospective monitoring of time-to-communication to complete the risk management cycle.

## Data Availability

The data generated and analyzed in the presented study are available from the corresponding author upon request.
